# NCL Inhibition Exerts Antineoplastic Effects against Prostate Cancer Cells by Modulating Oncogenic MicroRNAs

**DOI:** 10.3390/cancers12071861

**Published:** 2020-07-10

**Authors:** Tyler Sheetz, Joseph Mills, Anna Tessari, Megan Pawlikowski, Ashley E. Braddom, Tasha Posid, Debra L. Zynger, Cindy James, Valerio Embrione, Kareesma Parbhoo, Claudia Foray, Vincenzo Coppola, Carlo M. Croce, Dario Palmieri

**Affiliations:** 1 Department of Cancer Biology and Genetics, The Ohio State University Wexner Medical Center, Columbus, OH 43210, USA; tyler.sheetz@osumc.edu (T.S.); mills.647@buckeyemail.osu.edu (J.M.); anna.tessari@osumc.edu (A.T.); mpawlik15@gmail.com (M.P.); braddom@livemail.uthscsa.edu (A.E.B.); valerio.embrione@osumc.edu (V.E.); kareesmaparbhoo@yahoo.co (K.P.); claudia.foray@gmail.com (C.F.); vincenzo.coppola@osumc.edu (V.C.); carlo.croce@osumc.edu (C.M.C.); 2 Comprehensive Cancer Center, The Ohio State University, Columbus, OH 43210, USA; 3 Department of Urology, The Ohio State University Wexner Medical Center, Columbus, OH 43210, USA; tasha.posid@osumc.edu; 4 Department of Pathology, The Ohio State University Wexner Medical Center, Columbus, OH 43210, USA; debra.zynger@osumc.edu; 5 Mass Spectroscopy and Proteomics Facility, The Ohio State University, Columbus, OH 43210, USA; biokmst1@gmail.com

**Keywords:** nucleolin, microRNAs, prostate cancer, castration-resistant prostate cancer

## Abstract

Prostate cancer (PCa) is the most frequently diagnosed cancer in men and second most common cause of cancer-related deaths in the United States. Androgen deprivation therapy (ADT) is only temporarily effective for advanced-stage PCa, as the disease inevitably progresses to castration-resistant prostate cancer (CRPC). The protein nucleolin (NCL) is overexpressed in several types of human tumors where it is also mislocalized to the cell surface. We previously reported the identification of a single-chain fragment variable (scFv) immuno-agent that is able to bind NCL on the surface of breast cancer cells and inhibit proliferation both in vitro and in vivo. In the present study, we evaluated whether NCL could be a valid therapeutic target for PCa, utilizing DU145, PC3 (CRPC), and LNCaP (androgen-sensitive) cell lines. First, we interrogated the publicly available databases and noted that higher NCL mRNA levels are associated with higher Gleason Scores as well as with recurrent and metastatic tumors. Then, using our anti-NCL scFv, we demonstrated that NCL is expressed on the surface of all three tested cell lines and that NCL inhibition results in reduced proliferation and migration. We also measured the inhibitory effect of NCL targeting on the biogenesis of oncogenic microRNAs such as miR-21, -221 and -222, which was cell context dependent. Taken together, our data provide evidence that NCL targeting inhibits the key hallmarks of malignancy in PCa cells and may provide a novel therapeutic option for patients with advanced-stage PCa.

## 1. Introduction

Prostate cancer (PCa) is the most prevalent type of cancer among men in the United States and accounts for roughly 10% of cancer-specific mortality [1]. While the majority of PCa cases are confined to the prostate, patients with metastatic disease at diagnosis display a 5-year survival rate of only 30% [1]. Although advanced stage PCa is initially treated effectively with androgen deprivation therapy (ADT), progression to castration-resistant prostate cancer (CRPC) usually develops about 1 year after initiation of ADT [2]. Based on recent data, CRPC is becoming increasingly prevalent, with an average of 2–4% of PCa cases progressing to CRPC each year [3].

To achieve a CRPC phenotype, PCa cells evade ADT by acquiring aberrations in the androgen receptor (AR) pathway [4,5]. Overall, more than 70% of metastatic CRPC cases exhibit at least one AR pathway aberration [6], with over 50% of these tumors harboring a mutated or amplified AR gene [7].

Recent therapeutic approaches combating mechanisms of castration resistance have produced only modest survival benefits [4], and patients typically succumb to their disease within 9–30 months after progression to CRPC [3]. Conventional therapies for PCa are still associated with significant side effects [8], and trials for targeted therapies have been largely ineffective [9]. Thus, there remains a substantial unmet need for more effective therapies.

MicroRNAs (miRNAs or miRs) are deregulated in most types of cancer [10,11], and are emerging for their utility as potential biomarkers [12,13,14,15]. However, targeting miRNAs in applications for cancer therapy has proven more challenging, especially due to the lack of cancer cell-specific targeting and drug delivery [16,17].

A protein involved in miRNA regulation, nucleolin (NCL), is expressed predominantly in the nucleolus in normal cells [18] and is known to participate in a multitude of different cellular processes [19,20,21]. In cancer cells, NCL becomes aberrantly overexpressed [22,23], translocated to the cell membrane [24,25], and participates in several cancer-driving activities [26,27,28,29,30,31] including biogenesis of oncogenic miRNAs such as miR-21, -103, -221, and -222 [32]. NCL has been shown to exhibit similar cellular behavior in PCa, and exhibits greater surface expression in CRPC cell lines than in hormone-naïve models [33]. 

Although NCL-dependent miRNA regulatory activity is not limited to miR- 21, -221, and -222, these molecules have been profiled extensively in the literature for their role in most neoplasms, including PCa. miR-21 is overexpressed in the majority of solid tumors and it is addictive to cancer cells, leading to withdrawal-induced tumor regression [34]. There is mounting evidence for miR-21 implication in the pathogenesis and progression of chemo-resistant [14,35,36], castration-resistant [37,38], and clinically aggressive PCa [15,39,40]. 

Also regulated by NCL, miR-221/222 promote prostate cancer cell proliferation [41,42], and confer resistance to apoptosis [43]. Additionally, miR-221/222 are involved in AR functional integrity [44] and signaling pathway [45], thus have been implicated in the progression to CRPC [46,47,48]. Taken together, these data provide a compelling case for the use of miR-221/222 inhibitors in concert with miR-21 inhibitors for both hormone-naïve and CRPC therapy.

Given the association of NCL-dependent miRNAs with cancer, and that NCL expression increases in malignant cells where it is aberrantly expressed on the cell surface, we developed a therapeutic anticancer agent to bind NCL and block its miRNA biogenesis activity. This agent, named 4LB5, is a ~27 kDa single chain Fragment-variable (scFv) antibody that binds to the RNA-binding domain of NCL on the cell surface. 4LB5 becomes efficiently internalized, disrupts the biogenesis of miRs -21, -103, -221, and -222, induces apoptosis, decreases proliferation, and reduces tumor burden in vivo in breast cancer models [49].

In this study, we aim to expand the use of the scFv 4LB5 to both hormone-sensitive and CRPC cell lines, with a secondary aim to study its effects on AR expression. We show that 4LB5 is able to bind NCL on the surface of PCa cell lines and exert its intracellular effects on miRNA expression levels. Additionally, we provide evidence that 4LB5 can halt prostate cancer cell proliferation and migration and influence the CRPC cell phenotype at nanomolar concentrations. 

## 2. Results

### 2.1. NCL is Upregulated in Aggressive Forms of PCa

**Figure 1:**
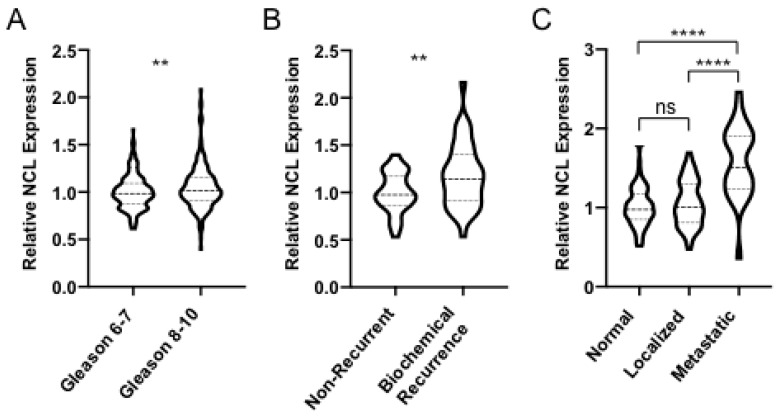
NCL is Upregulated in Aggressive Forms of PCa Violin plots displaying nucleolin (NCL) mRNA expression levels in patient tumor samples stratified by clinical characteristics from (**A**) TCGA provisional database, queried through cBioPortal (Gleason 6–7 *n* = 291; Gleason 8–10 *n* = 206; Pearson: r = 0.134; Spearman: rs = 0.127), and (**B**) Sun et al. (non-recurrent *n* = 39; biochemical recurrence *n* = 38), (**C**) Yu et al., and Chandran et al. queried through NCBI GEO (normal tissue *n* = 81; localized prostate cancer (PCa) *n* = 65; metastatic PCa *n* = 25 sample locations from four patients). A Student’s t-test was used for group analyses. ** *p* < 0.01, **** *p* < 0.0001.

We began our study by querying three publicly available gene expression databases containing PCa sets with different stages of diseased and normal tissue. We observed a statistically significant positive linear correlation between Gleason Score (GS) and whole-cell NCL expression (Pearson: r = 0.134, *p* = 0.003; Spearman: rs = 0.127, *p* = 0.005). Additionally, we found a statistically significant difference in NCL expression levels in GS = 6–7 tumors compared with GS = 8–10 tumors (*p* = 0.0039, Figure 1A). Further investigation also revealed significantly elevated NCL levels in recurrent disease compared to primary (*p* = 0.0031, Figure 1B) and metastatic disease compared to primary tumors and normal tissue (*p* = 2.6 × 10^−9^ and *p* = 2.3 × 10^−13^, Figure 1C). This analysis revealed that the NCL transcript is overexpressed in clinically advanced tumors, in agreement with previous reports [33,50,51]. No significant correlation between NCL levels and Overall Survival (OS) was identified in our bioinformatics analysis with additional data. 

### 2.2. 4LB5 Binds to PCa Cells and Inhibits Cell Proliferation

**Figure 2:**
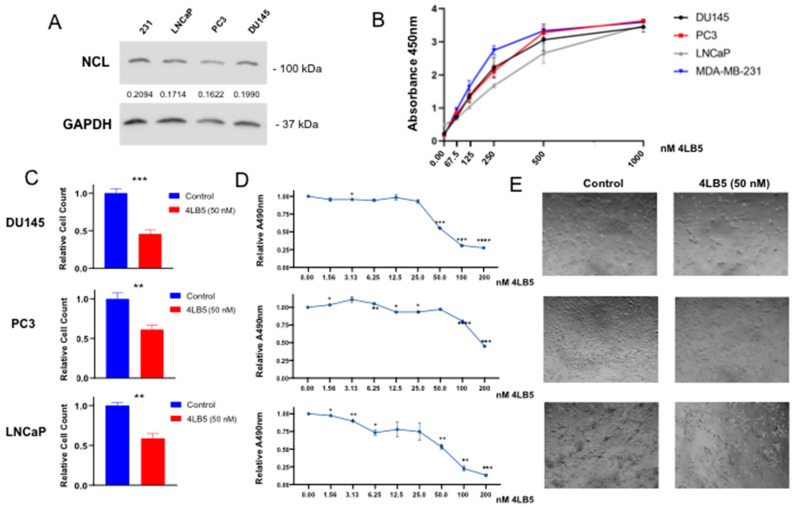
4LB5 Binds to PCa Cells and Inhibits Cell Proliferation (**A**) Basal expression levels of whole-cell NCL protein was measured in LNCaP, PC3, and DU145 PCa cell lines and compared to MDA-MB-231 expression levels via Western blot. Uncropped blots of Figure 2A are shown in Figure S4. (**B**) 4LB5 binding cell surfaces was assessed by an ELISA performed after incubating cells with serial dilutions of 4LB5. ELISA data shown are representative of two independent experiments performed in quadruplicate. (**C**) Relative cell counts of DU145, PC3, and LNCaP cells treated with 50 nM 4LB5 or control solution for 48 h. Cell survival data are the average of five biological replicates. (**D**) MTS assay performed after 72 h-treatment with increasing concentrations of 4LB5. MTS data are average of two assays performed in biological triplicate. (**E**) Light microscopy images of PCa cells taken at 48 h post-treatment with 50 nM 4LB5 or control solution. * *p* < 0.05, ** *p* < 0.01, *** *p* < 0.001, **** *p* < 0.0001.

Next, we selected three cell lines commonly used as in vitro models of advanced stage PCa and measured whole-cell NCL by Western blot. All lines showed a robust expression of NCL protein ranging from about 80–95% of the amount found in MDA-MB-231 cells used as a reference (Figure 2A). To assess NCL cell surface expression in DU145, PC3, and LNCaP, we then performed a cell surface ELISA using 4LB5 as the primary antibody. As seen in Figure 2B, significant binding of cell surfaces was already observed starting at 4LB5 concentrations approaching 62.5 nM. Notably, we found that LNCaP cells required higher concentrations of 4LB5 than PC3 and DU145 to show detectable binding. Similar binding curves were observed using MDA-MB-231 as positive control.

Next, we sought to determine whether 4LB5 inhibited the cell proliferation of PCa cells, as exhibited in other cancer cell types [49]. We exposed PCa cells to 50 nM 4LB5 or control solution. Cell counts of all three lines at 48 h after treatment were decreased by 30–60% compared to controls (Figure 2C). We also performed MTS assays with serial dilutions of 4LB5. A significant decrease in cell metabolic activity was seen at concentrations as small as 3.125 nM for DU145 and LNCaP cells (Figure 2D), with calculated IC_50_ values of 46.0 and 66.7 nM, respectively (Figure S1). This inhibitory effect was also evident upon microscopic examination of all tested cell lines treated with 4LB5 for 48 h (Figure 2E), 72 h, and 96 h (Figure S1B), though PC3 cells displayed a more 4LB5-resistant phenotype. Altogether, these data indicate that 4LB5 is effective in inhibiting PCa cell proliferation at nanomolar concentrations.

### 2.3. Treatment with 4LB5 Reduces Mature Forms of Oncogenic MicroRNAs and Initiates Apoptosis

**Figure 3:**
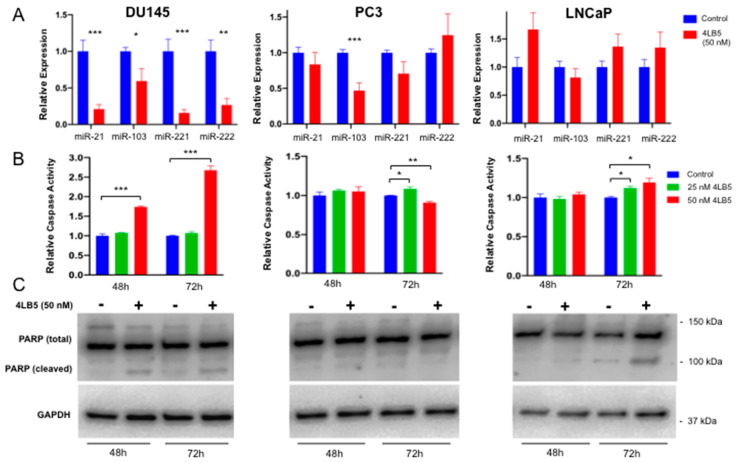
Treatment with 4LB5 Reduces Mature Forms of Oncogenic MicroRNAs and alters cell survival (**A**) microRNA expression levels were assessed via qRT-PCR in DU145, PC3, and LNCaP cells after 48 h treatment with 50 nM 4LB5 or control solution. qRT-PCR data are the average of three independent experiments performed in at least technical duplicate. (**B**) Caspase 3/7 activity was assessed after 48 and 72 h of 4LB5 treatment. Caspase assay was performed in biological triplicate for each time point and averaged. (**C**) PARP cleavage was assessed by Western blot after PCa cell treatment with 4LB5 or control solution for 48 and 72 h. PARP Western blots are representative of at least two independent experiments. Student’s t-test (2-tailed, homoscedastic) was utilized for statistical analysis. Uncropped blots of Figure 3C are shown in Figure S4. * *p* < 0.05, ** *p* < 0.01, *** *p* < 0.001, **** *p* < 0.0001.

We have previously shown that 4LB5 gets internalized into breast cancer cells causing reduction in miR-21, miR-103, miR-221, and miR-222 and initiating apoptosis [49]. To test this we treated PCa lines with 50 nM 4LB5 and measured the effects on mature miRNA levels and markers of apoptosis. As seen in Figure 3A, 4LB5 treatment resulted in >50% statistically significant reduction in all tested miRNAs in DU145 cells. A similar but less-pronounced effect was observed in PC3 cells. In LNCaP cells this effect was not seen with any of the tested miRNAs (Figure 3A). Upon 4LB5 treatment, primary forms of miR-21, -221 and -222 (pri-miR-21, pri-miR-221 and pri-miR-222) accumulated in DU145 cells, further supporting that NCL inhibition prevents microRNA biogenesis (Figure S2B). Mild or no effects were observed in PC3 and LNCaP cell lines, which indicates that 4LB5 does not affect miRNA levels at the transcriptional level. The only exception in this regard is pri-miR-222 in PC3 cells. However, since pri-miR-221 and -222 are transcribed as the same long primary transcript, it is possible that this observation is related to technical differences in the probes used to evaluate these pri-miRNAs.

Next, we evaluated apoptosis by treating PCa cells for 48 h and 72 h with 50 nM 4LB5 and performing a Caspase 3/7 activation assay. A statistically significant increase in caspase activity was observed in DU145 cells at 48 h (*p* = 9.9 × 10^−5^, Figure 3B). Both DU145 and LNCaP samples achieved a statistically significant caspase activity at 72 h post-treatment (*p* = 6.8 × 10^−5^, *p* = 0.013). However, PC3 exhibited only a modest increase in caspase activity when treated with 25 nM 4LB5 and showed an opposite effect when treated with 50 nM 4LB5 (Figure 3B). Consistent with the caspase assay, treatment with 50 nM 4LB5 resulted in PARP (Poly-(ADP-ribose) polymerase) cleavage in DU145 and LNCaP treated cells at 48 h and 72 h, but not in PC3 (Figure 3C). 

Taken together, these results show that 4LB5 can successfully inhibit the microRNA biogenesis function of NCL and induce apoptosis in a cell line-dependent context.

### 2.4. 4LB5 Impairs PCa Cell Migration

**Figure 4:**
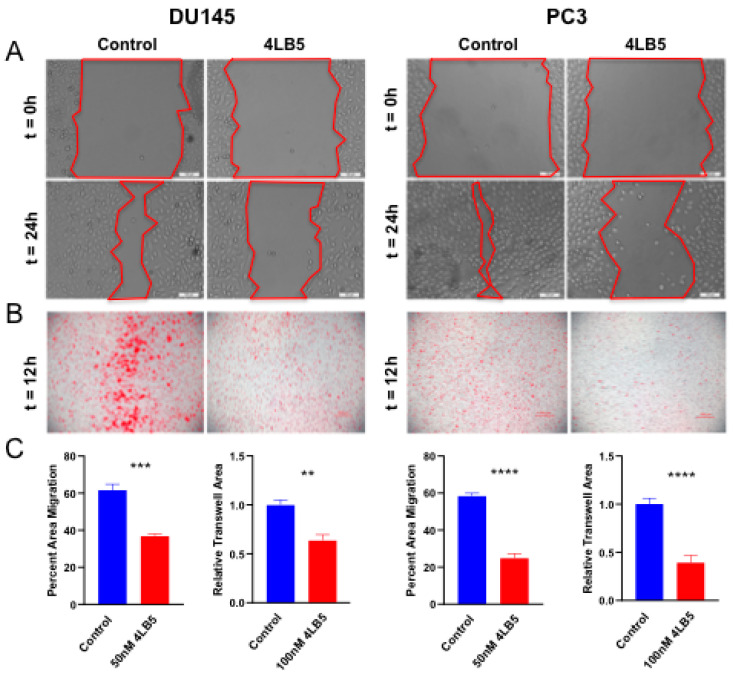
4LB5 Impairs PCa Cell Migration (**A**) Wound healing assays performed with DU145 and PC3 cells after 48h of pretreatment with 50 nM 4LB5 or control solution. Representative photos were taken at t = 0h and t = 24h. (**B**) Trans-well migration experiments performed with DU145 and PC3 cells after 24h pretreatment with 100 nM 4LB5 or control solution and 12h migration time. Migrated cells were stained with crystal violet and photographed with phase contrast microscopy. (**C**) Images of control- and 4LB5-treated migrated cells for experiments shown in A and B were processed and analyzed with ImageJ software in parallel. Quantification of images from five independent wound healing experiments performed in technical triplicate is reported. Trans-well migration data represents average of two independent experiments performed in biological triplicate and was generated by calculating percent area of migrated cells. ** *p* < 0.01, *** *p* < 0.001, **** *p* < 0.0001.

Since the NCL-dependent microRNAs are involved in epithelial to mesenchymal transition (EMT) phenotype in PCa [14,38,39,43,44,45,47,52,53,54,55], we tested the effects of 4LB5 treatment on cell migration. We did not include LNCaP cells in these experiments due to their weak adherence to culture vessels and tendency to dissociate spontaneously [56]. We pretreated DU145 and PC3 cells with 4LB5 or control solution before performing wound healing assay and measuring percent area migration after 24 h. As seen in both cell lines in Figure 4A, migration fronts advanced visibly less after 24 h in the 4LB5-treated condition compared with the control. The average percent area migration of control- and 4LB5-treated cells was 59% and 25% for PC3 (*p* = 2.8 × 10^−6^), and 62% and 37% for DU145 (*p* = 1.0 × 10^−4^), respectively (Figure 4C). Similarly, trans-well migration assays were performed on cells pretreated with 4LB5 or control solution. After 12 h incubation, images were acquired (Figure 4B) and processed with ImageJ software, and percent area of each trans-well membrane covered by migrated cells was calculated (Figure 4C). 4LB5-treated DU145 cells migrated 36% less than control treated cells (*p* = 8.1 × 10^−4^), and 4LB5-treated PC3 cells migrated 61% less than their control counterparts (*p* = 1.1 × 10^−4^). In summary, Figure 4 illustrates that 4LB5 impairs migration in aggressive PCa cell lines.

### 2.5. 4LB5 Reduces AR Expression in Hormone-Sensitive LNCaP Cells

**Figure 5:**
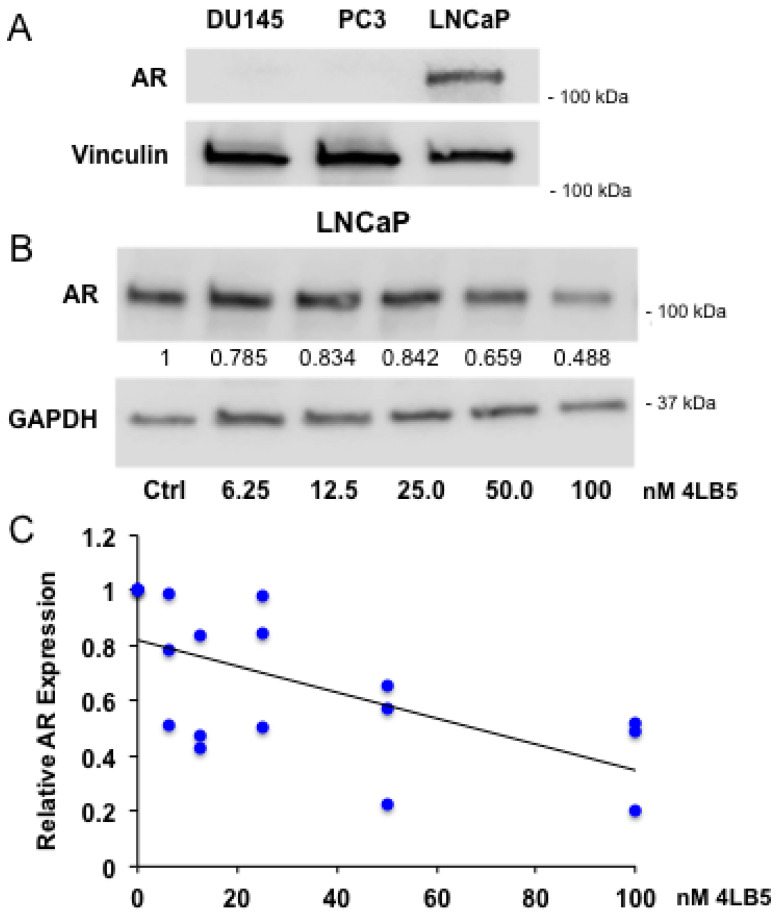
4LB5 Reduces AR Expression in Hormone-Sensitive LNCaP Cells (**A**) Basal androgen receptor (AR) expression in PCa cell lines. (**B**) LNCaP cells were treated with the indicated increasing concentration of 4LB5 for 48h and AR expression was assessed by Western blot. Additional loading control using Ponceau S staining is included in Figure S3A. (**C**) Levels of AR were normalized to GAPDH (Glyceraldehyde 3-phosphate dehydrogenase), plotted, and subjected to linear correlation testing (Pearson (displayed): r = −0.621, *p* = 0.006, Spearman: rs = −0.650, *p* = 0.003). Plotted data are from the experiment shown in Figure 5B as well as two additional independent experimental replicates.

Our findings indicate that 4LB5 inhibits LNCaP proliferation seemingly independently of NCL-specific miRNAs. Knowing that NCL is a multifunctional protein, and that previous groups demonstrated therapeutic efficacy of NCL-binding molecules independently of miRNAs [51,57,58,59], we hypothesized that 4LB5 could confer an inhibitory effect on AR expression. After verifying AR expression in LNCaP cells (Figure 5A), we treated them with serial dilutions of 4LB5 and quantified levels of AR post-treatment. We observed a statistically significant negative linear correlation between 4LB5 dose and AR expression (Pearson: r = −0.621, *p* = 0.006, Spearman: rs = −0.650, *p* = 0.003), with a 51% reduction in AR expression in cells treated at the highest dose compared with controls (Figure 5B). These data demonstrate that 4LB5 decreases AR protein levels in AR-positive prostate cancer cells.

## 3. Discussion

Despite mounting evidence that NCL is a valid therapeutic target in different types of aggressive cancer [29,32,57,59,60], the data for PCa are lacking. In the present study we aimed to demonstrate that targeting surface NCL by the scFv 4LB5 is an effective way to inhibit multiple neoplastic features of PCa cells.

First, we took advantage of publicly available databases to perform an analysis of human PCa tumors and validate the expression levels of NCL. Overall we found a statistically significant positive association between NCL transcript levels and clinically aggressive tumors. As expected, this difference was more pronounced in metastatic prostate cancer tissue compared to primary disease or normal tissue (Figure 1C). Admittedly, we are unable to draw conclusions about whole cell protein or surface protein levels with this approach.

Using ELISA, we then show that 4LB5 efficiently binds PCa cell surface NCL in vitro, consistent with the previous literature [33,50,51,61]. We selected three different cell lines to reasonably represent the cellular heterogeneity typical of advanced disease [4,6,7,9] and unsurprisingly observed different degrees of antineoplastic effects upon treatment with 4LB5. We observed that androgen-dependent LNCaP cells show the least avid binding to NCL (Figure 2B), aligning with previous studies reporting higher surface NCL expression in CRPC phenotypes [33]. Furthermore, the LNCaP cell line often clumps at high densities and fails to form a cell monolayer [56] likely preventing complete exposure of the cell surface to antibodies.

4LB5 reduced PCa cell survival, proliferation and migration, and induced apoptosis, which is consistent with our previous work [49]. Of note, since the amount of detected PARP cleavage was limited, it is possible that the observed phenotypes are due to a combination of multiple factors, such as inhibition of cell proliferation and concurrent activation of alternative cell death pathways. However, although 4LB5 had a statistically significant inhibitory effect on the proliferation of all cell lines, PC3 cells exhibited the greatest resistance in these experiments (Figure 2, Figure S1). While 4LB5 bound avidly to PC3 cells, downregulation of NCL-dependent microRNAs was variable, caspase activation was similar to controls, and PARP cleavage was not observed. The reduced apoptotic response can be expected in PC3 cells because they are p53-negative [62]. However, the lack of p53 would not explain the absence of miRNA downregulation after NCL inhibition. Therefore, it is conceivable that PC3 cells have additional intrinsic resistance due to mechanisms involving the molecular steps between 4LB5 binding to NCL on the cell surface and the microRNA biogenesis. Notably, PC3 cell proliferation and migration were significantly impaired by 4LB5 treatment, indicating that this resistance does not encompass all phenotypic effects.

Regarding apoptosis, DU145 cells exhibited the strongest response to 4LB5 followed by LNCaP, and finally PC3. Interestingly, LNCaP cells appeared to activate apoptosis independently of miRNA inhibition. Although this study investigated only apoptosis, it is likely that NCL inhibition portends other mechanisms of cell death [58,59,63] given NCL’s multiple roles in cancer [23].

We have previously shown that 4LB5 can suppress NCL-dependent miRNA biogenesis in breast cancer cells [49]. In this study, we report a similar effect in DU145 cells. NCL-dependent miRNA levels in PC3 cells were too variable to attain statistical significance (Figure 3A). One possible contributing factor is substantial post-treatment cell death. In LNCaP cell lines, we believe that we didn’t observe a measurable effect by qRT-PCR in part because basal miRNA expression is extremely low (Figure S2). Notably, NCL belongs to a class of RNA-binding proteins (RBPs) able to modulate the biogenesis of a specific microRNA subset [32]. It is tempting to speculate that multiple factors affect the ability of NCL to recognize RNA, including their tridimensional structure or the presence of co-factors or other RBPs which could cooperate with NCL biological functions. Our work here further supports the idea that NCL-selective mechanisms of action in RNA biology require extensive further studies. 

4LB5 has the unique ability to specifically recognize the NCL RNA-binding site and interfere with miRNA biogenesis, but it also inhibits the general oncogenic functions of NCL. In this regard, we were intrigued that 4LB5 can suppress proliferation of the androgen responsive LNCaP cells independently of NCL-dependent miRNAs. This led us to hypothesize that other mechanisms, possibly related to the AR expression and signaling pathway, could be responsible for the anti-proliferative effect of 4LB5 in this setting. In line with our hypothesis, we found that 4LB5 concentration inversely correlated with AR protein expression (Figure 5). Therefore, we can reasonably conclude that the inhibition of NCL would be beneficial both in androgen-dependent and androgen-independent cells by virtue of at least two independent mechanisms. In androgen-dependent PCa cells, we can speculate that NCL inhibition by 4LB5 reduces AR expression and, in turn, induces cell death. However, in AR-negative CRPC lines such as DU145 that overexpress NCL-dependent miRNAs, suppression of these miRNAs by NCL inhibition seems sufficient to induce apoptosis, which is a previously established mechanism [34]. 

4LB5 assertion of its antineoplastic effects via multiple mechanisms is another motivating factor that warrants further development of this scFv antibody. 4LB5 would offer new potential clinical options in the CRPC setting, for which new therapies are still needed. Future studies should focus on pharmacokinetic and pharmacodynamic testing in in vivo PCa models. 

Additional research should also include the investigation of the mechanism by which 4LB5 downregulates AR. Previous studies have suggested a link between miR-21 and AR [37,38], though miR-21 was not affected in our experiments upon treatment with 4LB5 in LNCaP cells, suggesting still an additional mechanism at play. 

Lastly, given its recombinant nature and inherent versatility [64], 4LB5 can be conjugated to other molecules or protein domains to boost its clinical efficacy (e.g., adding immunoreactive Fc domain or active RNAse enzyme [65]). In fact, scFv antibodies are currently being investigated in PCa to target cell surface molecules such as PSMA for diagnostic purposes [66,67]. The ability to bind a surface protein highly expressed in PCa makes 4LB5 a powerful potential theranostic tool.

In summary, here we show that NCL might play a role in human prostate tumorigenesis through cell-type specific mechanisms that involve both previously identified and novel NCL-dependent microRNAs. Most importantly, we provide evidence in support of innovative therapeutic strategies for the treatment of PCa based on the use of anti-NCL immuno-agents, which can simultaneously accomplish the inhibition of the AR pathway. Hence, our results pave the way for further studies that will be required both for the validation of anti-NCL targeting strategies specific to neoplastic prostate cells and for the dissection of the mechanisms linking NCL to the AR pathway.

## 4. Materials and Methods 

### 4.1. Cell Culture and Media

LNCaP (androgen-dependent), DU145 and PC3 (androgen-independent), and MDA-MB-231 (breast cancer positive control) cells were cultured in RPMI media containing 10% Fetal Bovine Serum and 1% penicillin/streptomycin in a humidified atmosphere at 5% CO_2_ and 37 °C [62].

### 4.2. 4LB5 Production, Purification, and Treatments

4LB5 scFv was expressed, extracted, purified, and tested for quality as a recombinant protein by the Proteomics Shared Resource at the Ohio State University, as previously described [49]. Control (vehicle) solution was prepared with all components of 4LB5 solution minus the scFv protein. For experiments involving treatment with 4LB5, an appropriate concentration was chosen to balance accomplishing the goals of the experiment and preventing excessive cell death due to 4LB5′s cytotoxic effects. 4LB5 (or an equivalent volume of control buffer) was added to cell media in a volume sufficient to produce the final desired concentrations.

### 4.3. Cell Surface ELISA

To determine the ability of 4LB5 to bind PCa cell surfaces, ELISA was performed as previously described [49]. Briefly, 5 × 10^4^ cells/well were cultured for 24 h in a 96-well plate. Serial dilutions of 4LB5 were incubated with cells for 2 h at room temperature. Cells were washed with PBS and incubated with horseradish peroxidase (HRP)-conjugated antipenta-His antibody (Qiagen, Germantown, MD, USA; 1:10,000 dilution per manufacturer’s instructions) for 1h at room temperature. Following additional washes, TMB reagent (Sigma, St. Luis, MO, USA) was added for 10 min before quenching with 1M HCl. Absorbance was read at 450 nm.

### 4.4. Cell Viability and Proliferation Assays

To evaluate the effect of 4LB5 on PCa cell viability, cells were cultured in 12-well plates for 24 h and treated with media containing 50 nM 4LB5 or control solution for additional 48 h. Surviving cells were counted using a hematocytometer.

To test the effects of 4LB5 on PCa cell proliferation, cells were cultured in a 96-well plate for 24 h and then treated with serial dilutions of 4LB5 (0–200 nM). After 48 h or 72 h, treatment media was replaced with media containing MTS (3-(4,5-dimethylthiazol-2-yl)-5-(3-carboxymethoxyphenyl)-2-(4-sulfophenyl)-2H-tetrazolium)) working solution and cells were incubated for 4 h at culture conditions. Absorbance was then read at 490 nm using a multi-well spectrophotometer (Molecular Devices, San Jose, CA, USA), with means plotted at each concentration, and utilized to construct a dose–response curve. IC_50_ values were calculated utilizing a 4-parameter logistic regression model with GraphPad Prism 8 (Figure S1A). IC_50_ was unable to be accurately calculated for PC3 due to inadequate number of data points at the lower plateau of the sigmoidal curve [68].

### 4.5. Western Blotting

For immunoblotting, cells were harvested and lysed in the presence of protease and phosphatase inhibitors as previously described [49]. Proteins were quantified using Bradford assay, diluted, and loaded with 5X Laemmli buffer into a 4–20% TGX gel. After electrophoretic separation, proteins were transferred to a nitrocellulose membrane. The membrane was stained with Ponceau S solution for equal loading control, an image was captured, and the membrane de-stained with TTBS. Membrane was blocked in 5% non-fat dry milk or 5% BSA in TTBS, and incubated with 1:1000–1:5000 dilution of Vinculin (Abcam, Cambridge, MA, USA), GAPDH-HRP, AR, NCL, or PARP (Cell Signaling, Danvers, MA, USA) antibodies overnight. Secondary antibody was added (if necessary) at 1:2500 dilution and membranes were visualized using HRP and a Li-Cor imaging system. Bands were quantified using Image Studio software (Li-Cor Biosciences, Lincoln, NE, USA) and normalized against housekeeping genes. Upon acquisition, images were exported as JPEG files before being assembled for figure preparation. Un-cropped original JPEG-exported images are reported in the Supplementary Material.

### 4.6. Quantitative Real-Time PCR

To assess microRNA and gene mRNA expression levels, the TaqMan Fast-PCR kit (Applied Biosystems, Foster City, CA, USA) was utilized according to the manufacturer’s instructions, followed by detection with the 7900HT Sequence Detection System (Applied Biosystems). All reactions were performed in technical triplicate. Simultaneous quantification of RNU6 and GAPDH was used as reference for miRNA and gene quantification, respectively. The comparative cycle threshold (Ct) method for relative quantification of gene and miRNA expression (User Bulletin #2; Applied Biosystems) was employed.

### 4.7. Caspase Activity Assay

To examine 4LB5 ability to induce apoptosis we utilized the Promega Caspase-Glo^®^ 3/7 Assay. Cells were cultured in a 96-well plate for 24 h and treated with media containing increasing concentrations of 4LB5. After 48 h or 72 h, caspase activity was assessed according to manufacturer’s instructions (Technical Bulletin #TB323; Promega, Madison WI, USA).

### 4.8. Trans-Well Migration and Wound Healing Assay

For trans-well migration, cells were grown to subconfluency, serum-starved and pretreated with 100 nM 4LB5 or control solution for 24h. Then, 1 × 10^5^ cells were seeded in 8-μm pore trans-well chambers (Greiner Bio-One, Monroe, NC, USA) in 24-well plates in serum-free medium plus 100 nM 4LB5 or control solution. Complete medium with 10% FBS was added to the bottom chamber and cells were incubated for 12 h. Trans-wells were then fixed and stained with crystal violet solution, and non-migrated cells removed. Images of the migrated cells were captured and color thresholded to obtain a binary image displaying cellular content as saturated black areas on a white background. Thresholded images were masked with ImageJ software to exclude non-cellular particles from the final area calculation as described previously [69].

For wound healing assays, 100% confluent cells on 12-well plates were pretreated with 50 nM 4LB5 or control solution for 48 h. Scratches were made with a sterile 200uL pipette tip. At least 4 photographs per condition were obtained immediately after scratching (t = 0) and again at t = 24 h. Area not covered by the migrated cell front was calculated at each time point.

### 4.9. Bioinformatics Analysis

To assess the relationship between NCL levels and tumor clinical characteristics, the TCGA dataset (TCGA Research Network: http://cancergenome.nih.gov/) was queried through cBioPortal; Sun et al., Yu et al., and Chandran et al. datasets were queried through NCBI GEO [70,71,72]. mRNA expression data corresponding to tumor clinical and pathological characteristics were downloaded through the respective platform and analyzed as indicated.

### 4.10. Statistical Analysis

A Student’s t-test (1-tailed, homoscedastic) was used (unless otherwise specified) to determine levels of significance between means in each experiment using a cutoff for statistical significance of *p* < 0.05. For qRT-PCR analysis, outliers with absolute or relative values +/− 2SD of the mean were excluded. Pearson and Spearman correlation coefficients were calculated (SPSS) to determine the relation of different variables in Figure 1A and Figure 5C, and nonlinear regression analysis (Prism 8, GraphPad Software, San Diego, CA, USA) in Figure S1A. Error bars in all figures represent +/−1 SE of the mean.

## 5. Conclusions

In this study, we provide evidence to support the use of 4LB5 to inhibit NCL and induce therapeutic responses in PCa cells. We also shed light on the previously unknown antiandrogenic activity of this molecule. These results support 4LB5 as a novel option for the treatment of PCa in its multiple clinical manifestations.

## Supplementary Materials

The following are available online at https://www.mdpi.com/2072-6694/12/7/1861/s1, Figure S1: Effects of 4LB5 on cancer cell proliferation; Figure S2: Effects of 4LB5 on microRNA biogenesis; Figure S3: Additional normalization of analysis performed in Figure 5; Figure S4: Uncropped Western Blot Figures.
