# Supplementary material for: NCL Inhibition Exerts Antineoplastic Effects against Prostate Cancer Cells by Modulating Oncogenic MicroRNAs
Source: Cancers (Basel). 2020 Jul 10;12(7):1861. doi: 10.3390/cancers12071861 (PMC7408652; doi:10.3390/cancers12071861)
Supplement: Supplementary file 1 [file cancers-12-01861-s001.zip › cancers-840010-supplementary final.docx]

Supplementary Materials

NCL Inhibition Exerts Antineoplastic Effects against Prostate Cancer Cells by Modulating Oncogenic MicroRNAs

Tyler Sheetz , Joseph Mills, Anna Tessari, Megan Pawlikowski, Ashley E. Braddom, Tasha Posid, Debra L. Zynger, Cindy James, Valerio Embrione, Kareesma Parbhoo, Claudia Foray, Vincenzo Coppola, Carlo M. Croce and Dario Palmieri


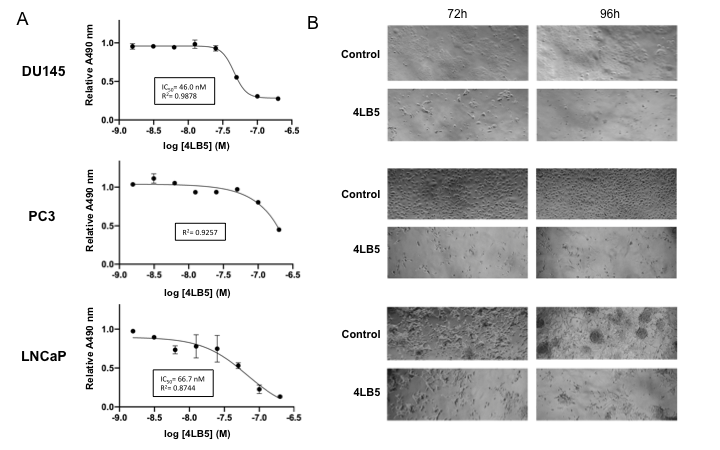


**Figure 1.** Effects of 4LB5 on cancer cell proliferation. (**A**) IC50 values calculated from fitted lines obtained by MTS assay performed after 72 h treatment with serial dilutions of 4LB5. MTS data are average of two assays performed in biological triplicate. (**B**) Phase contrast microscopy was utilized to obtain images of DU145, PC3, and LNCaP cells (top to bottom) obtained at 72 and 96 h post-treatment with 50 nM 4LB5 or control solution.


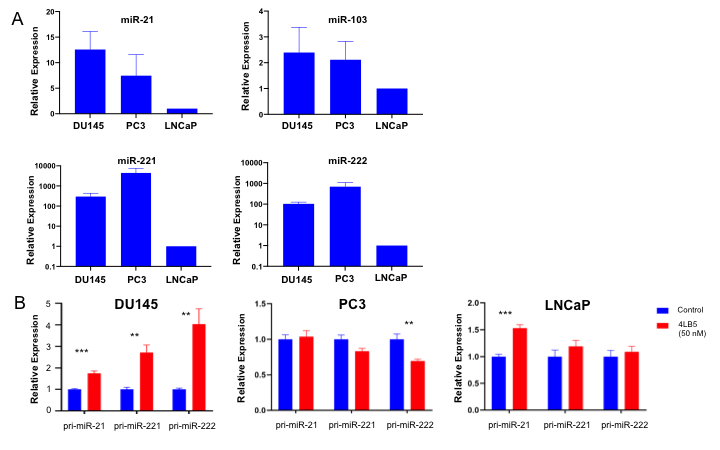


**Figure S2.** Effects of 4LB5 on microRNA biogenesis. (**A**) Basal expression levels of NCL-dependent microRNAs miR-21, miR-103, miR-221, and miR-222 quantified by qRT-PCR. Displayed values are relative to LNCaP expression values. Data represents the average of at least six independent experiments. (**B**) pri-miRNA expression levels were assessed via qRT-PCR in DU145, PC3, and LNCaP cells after 48hr treatment with 50 nM 4LB5 or control solution. qRT-PCR data are the average of two independent experiments performed in at least technical duplicate. Student’s t-test (2-tailed, homoscedastic) was utilized for statistical analysis. ***p* < 0.01, ****p* < 0.001.


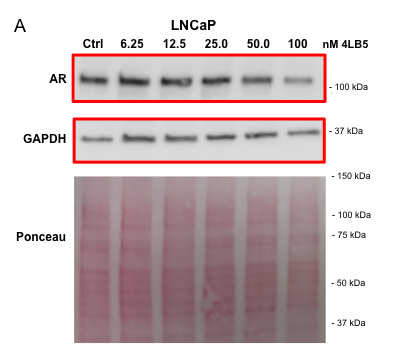


**Figure S3.** Additional normalization of analysis performed in Figure 5. LNCaP cells were treated with the indicated increasing concentration of 4LB5 for 48 h and AR expression was assessed by Western blot using GAPDH and Ponceau S staining to further assess even loading control. Blots indicated in red are the same as those shown in Figure 5B.
