# Supplementary figures and images for: NCL Inhibition Exerts Antineoplastic Effects against Prostate Cancer Cells by Modulating Oncogenic MicroRNAs
Source: Cancers (Basel). 2020 Jul 10;12(7):1861. doi: 10.3390/cancers12071861 (PMC7408652; doi:10.3390/cancers12071861)

Fig 2A - NCL

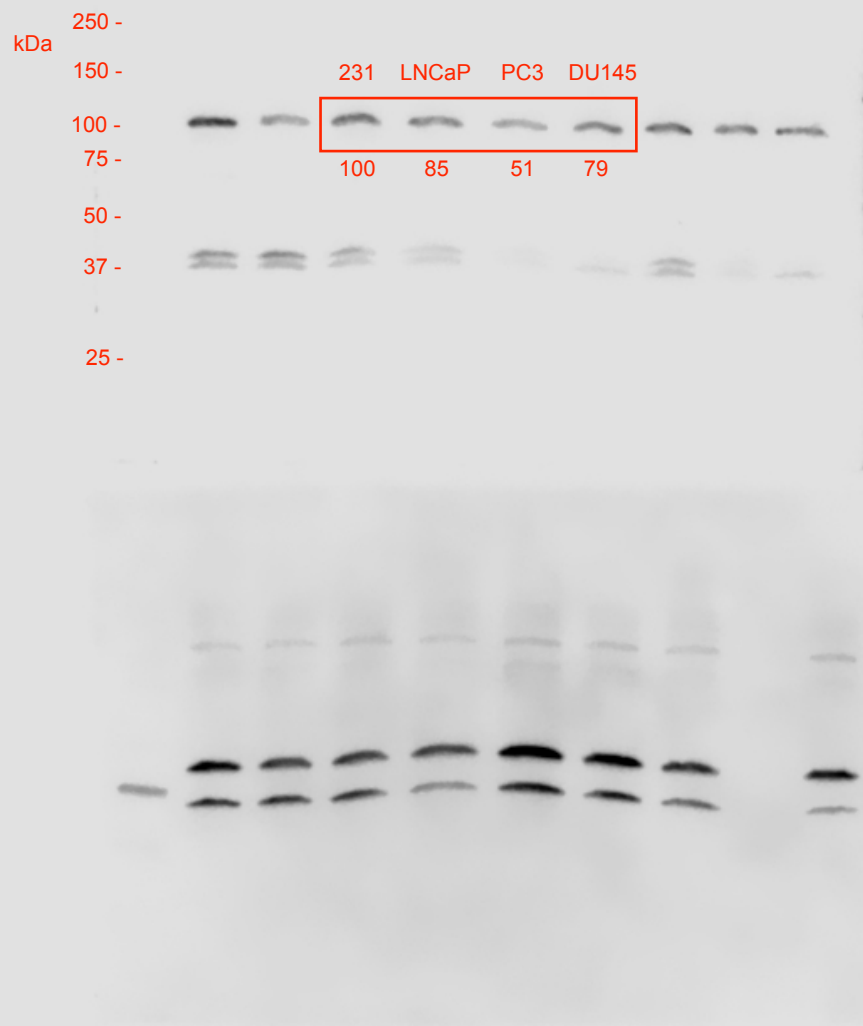

Fig 2A - NCL - ladder

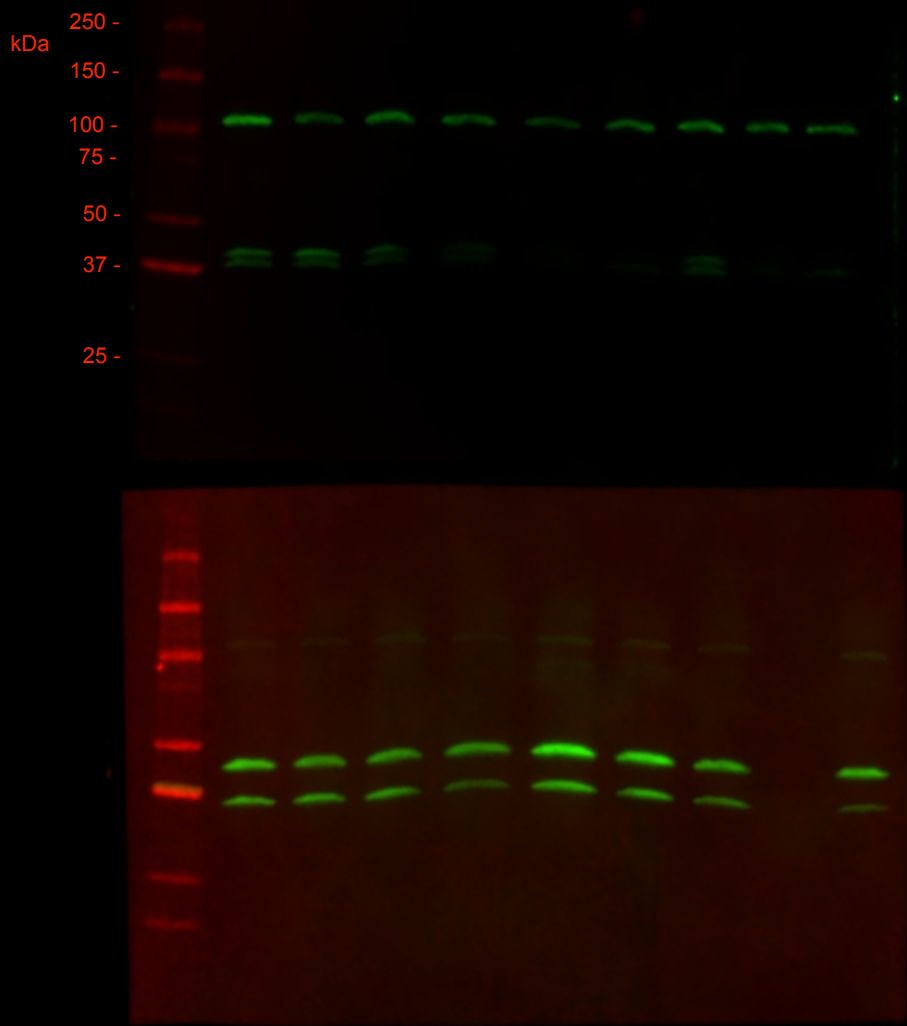

I

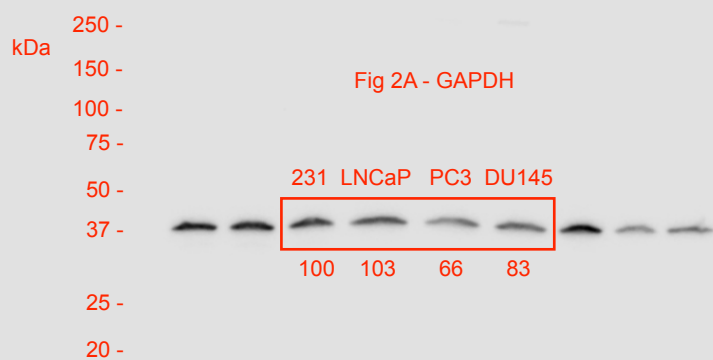

II

Fig 2A - GAPDH - ladder

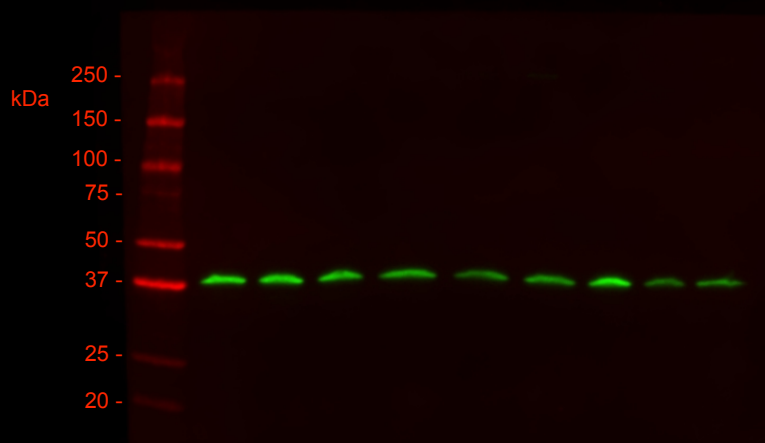

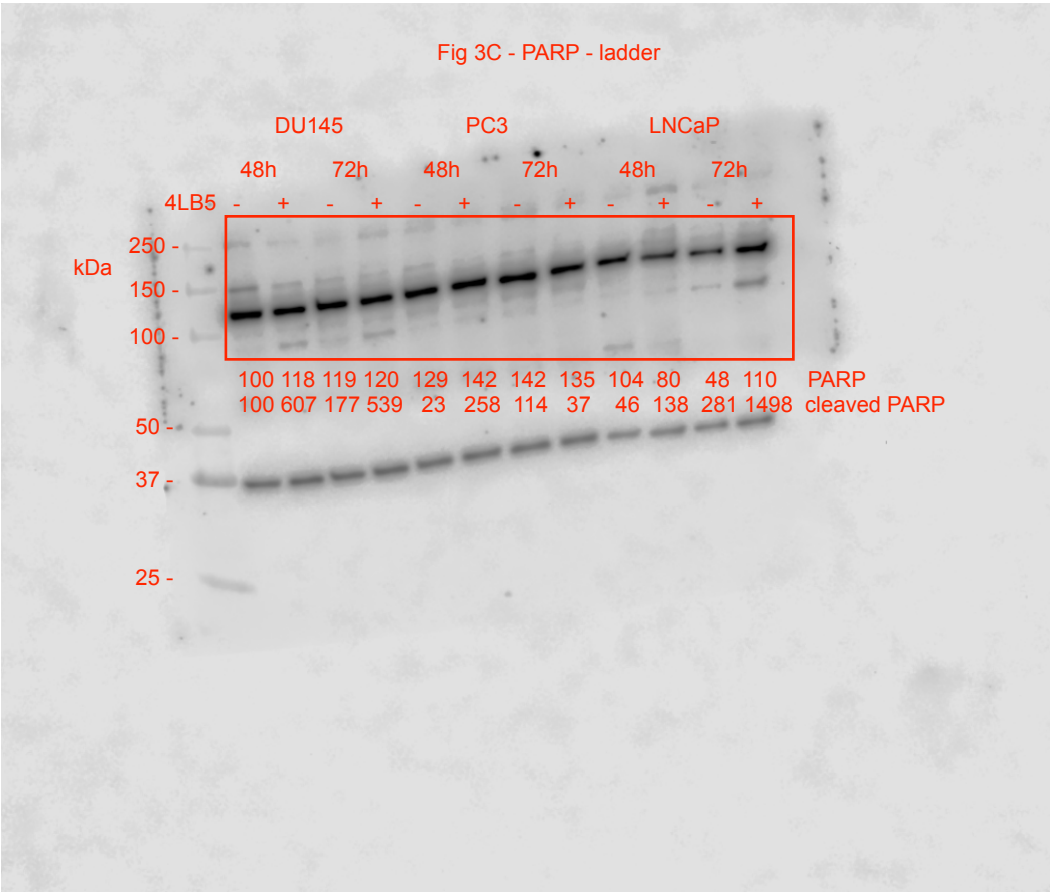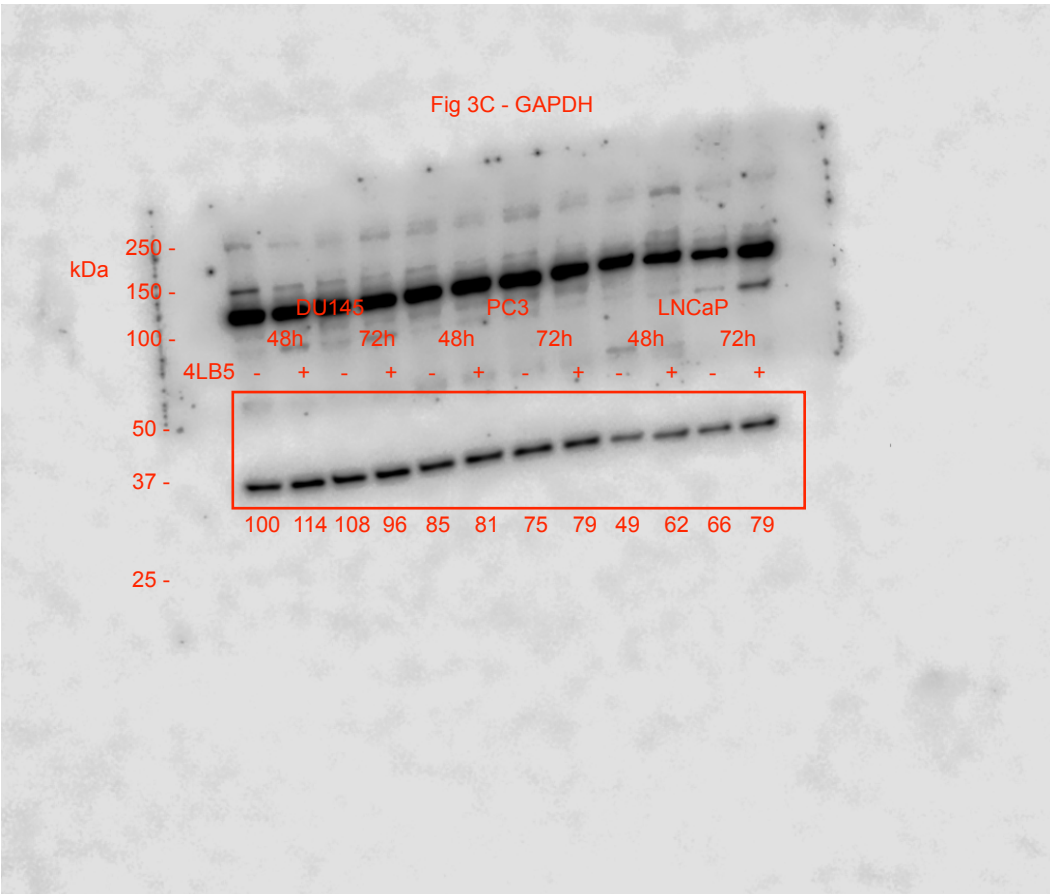

Fig 5A - AR

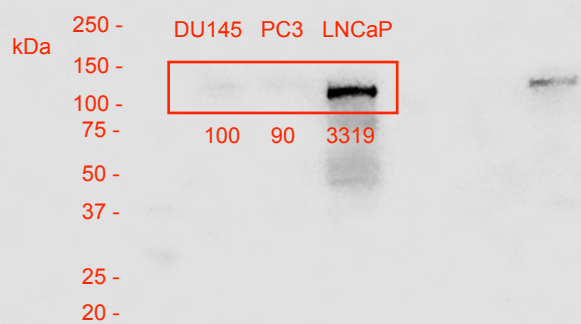

Fig 5A - AR - ladder

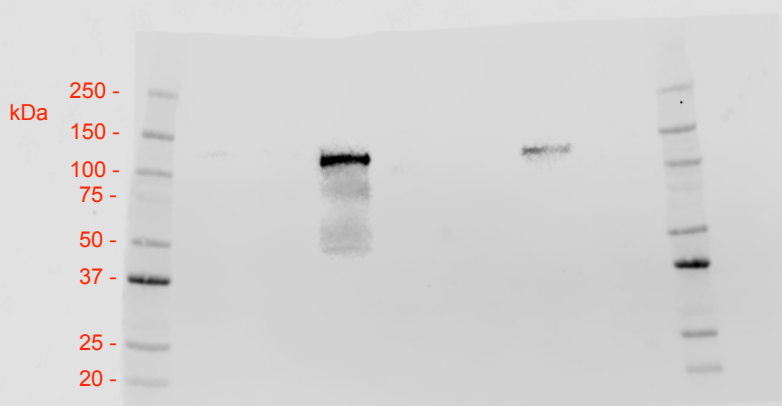

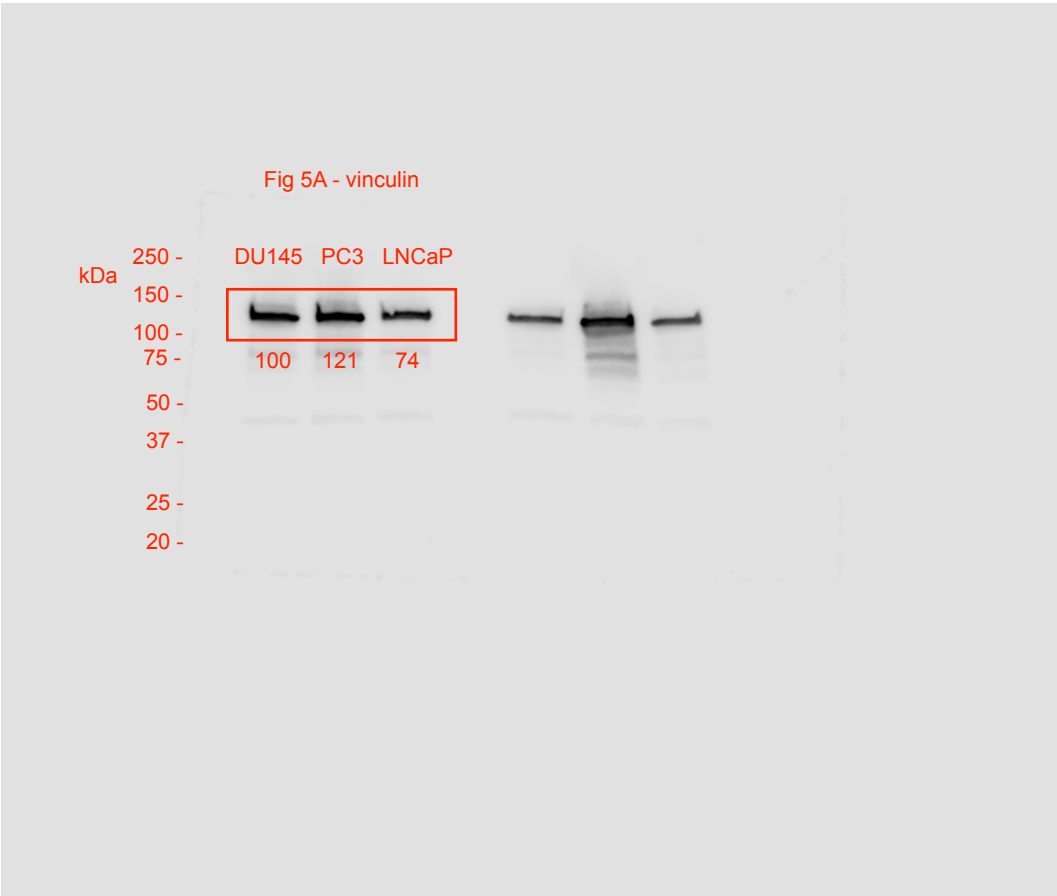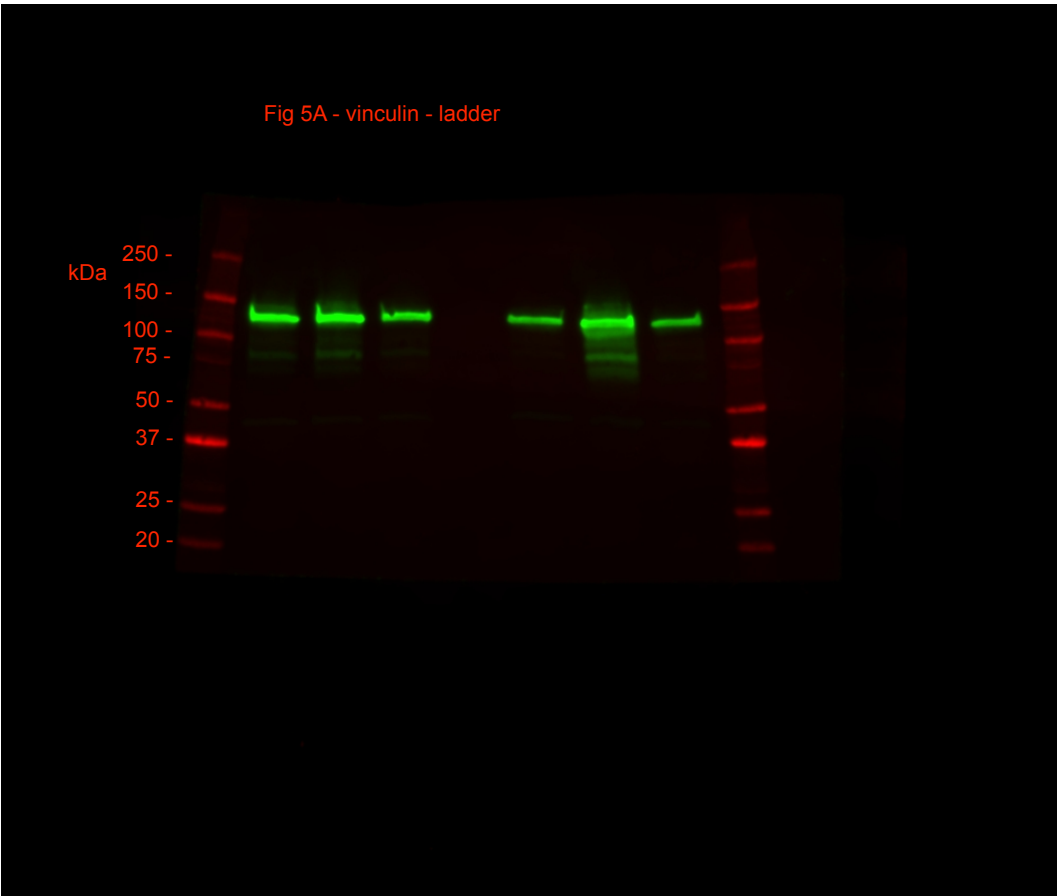

Fig 5B - AR

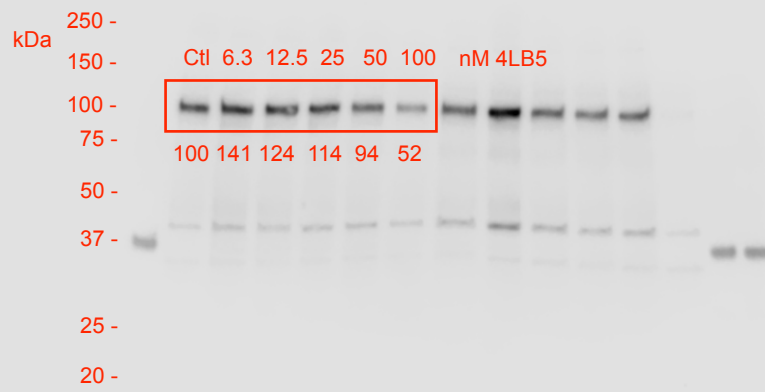

Fig 5B - AR - ladder

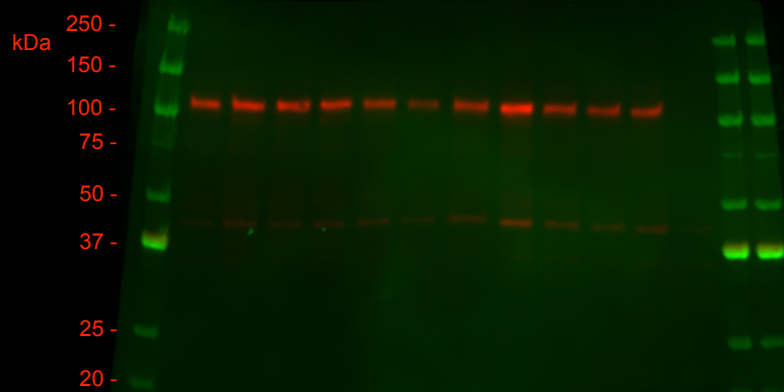

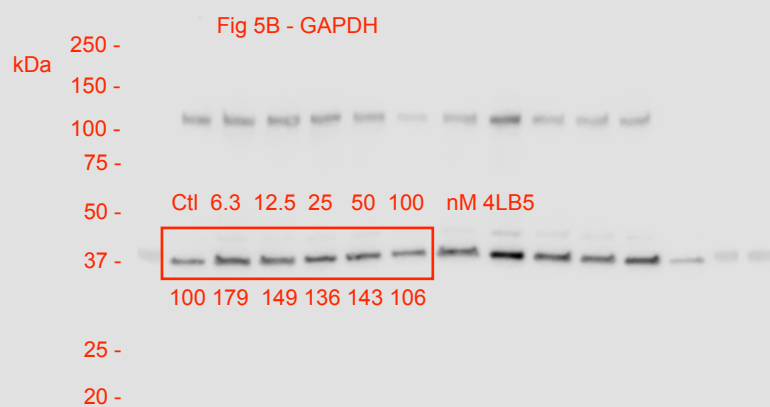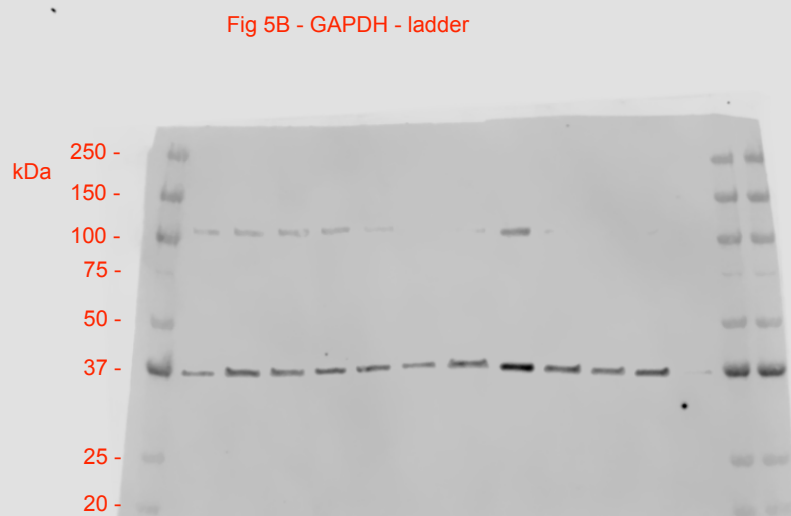

Supplement: Supplementary file 1 [file cancers-12-01861-s001.zip › cancers-840010 Figure S4. Uncropped Western Blot Figures.pdf]
